# Influence of Accidental Impurities on the Spectroscopic and Luminescent Properties of ZnWO_4_ Crystal

**DOI:** 10.3390/ma16072611

**Published:** 2023-03-25

**Authors:** Kirill Subbotin, Anatolii Titov, Victoria Solomatina, Andrew Khomyakov, Ekaterina Pakina, Viktor Yakovlev, Damir Valiev, Marina Zykova, Kristina Kuleshova, Yana Didenko, Denis Lis, Mikhail Grishechkin, Sergei Batygov, Sergei Kuznetsov, Igor Avetissov

**Affiliations:** 1 Prokhorov General Physics Institute of the Russian Academy of Sciences, 119991 Moscow, Russia; 2 Department of Chemistry and Technology of Crystals, Mendeleev University of Chemical Technology, 125047 Moscow, Russia; 3 Zelinsky Institute of Organic Chemistry of the Russian Academy of Sciences, 119991 Moscow, Russia; 4 School of Advanced Manufacturing Technologies, National Research Tomsk Polytechnic University, Lenin Avenue 30, 634050 Tomsk, Russia

**Keywords:** zinc tungstate, pure substance, inductively coupled plasma mass spectrometry, crystal growth, photoluminecense, cathodoluminecense

## Abstract

Special techniques for deep purification of ZnO and WO_3_ have been developed in this work. A ZnWO_4_ single crystal has been grown by the Czochralski method using purified ZnO and WO_3_ chemicals, along with the ZnWO_4_ crystal-etalon, which has been grown at the same conditions using commercially available 5N ZnO and WO_3_ chemicals. The actual accidental impurities compositions of both the initial chemicals and the grown crystals have been measured by inductively coupled plasma mass-spectrometry. A complex of comparative spectroscopic studies of the crystals has been performed, including optical absorption spectra, photo-, X-ray-, and cathodoluminescence spectra and decay kinetics, as well as the photoluminescence excitation spectra. The revealed differences in the measured properties of the crystals have been analyzed in terms of influence of the accidental impurities on these properties.

## 1. Introduction

The ZnWO_4_ single crystal has been well known for a long time. Its growth technology by Czochralski is well developed and allows obtaining large, high-quality boules [1,2,3,4,5]. This crystal has better thermal conductivity [1] and crack resistance [6,7] than many other tungstate and molybdate crystals. Despite low crystallographic symmetry [8], ZnWO_4_ crystal has very modest anisotropy of thermal expansion coefficients [1,9] that inhibits its thermo-mechanical strain during the crystal cooling after Czochralski growth [6].

Undoped ZnWO_4_ single crystals have their own strong and broadband fluorescence in the 380–650 nm spectral range under UV and other kinds of high-energy excitation [10], due to intra-center recombination of self-trapped excitons at the [WO_6_] cluster [11,12]. Therefore, ZnWO_4_ single crystals are promising for application as efficient scintillating materials [12,13,14,15]. Rare-earth doped ZnWO_4_ single crystals demonstrate very attractive spectroscopic properties and, thus, are the promising active media of diode-pumped solid-state lasers [16,17,18,19,20]. Some other applications of these crystals were demonstrated in Refs. [1,21].

The extremely high sensitivity of the optical and spectroscopic properties of ZnWO_4_ single crystal to some kinds of uncontrolled impurities has been well known for many years [7,20,22,23,24]. In particular, even very low concentrations of some impurities (1–5 ppm [10,11,25,26]) cause the broad and rather strong optical absorption bands in the crystal peaking near 325–350 nm, 430–450 nm and 500 nm. These absorption bands result in pink or brown coloration of the crystals. The first band is mainly attributed to Fe^3+^ ion, and the second one is attributed to the Fe^2+^ ion [2,10,22,24,26]. Concerning the attribution of the last band, there are several versions: the main ones are Cr^3+^ ion [22] or the color centers at oxygen vacancies [27].

The version of Cr^3+^ looks quite questionable in our opinion. In fact, according to the crystal field theory and numerous experimental studies of Cr^3+^ doped transparent materials, there should be two main broad optical absorption bands with comparable intensities. One should be the band peaking at 400–500 nm (depending on the particular crystal field strength) and corresponding to the ^4^A_2_→^4^T_1_ vibronic transition. Another one is peaking at 650–750 nm and corresponding to the ^4^A_2_→^4^T_2_ transition [28]. Moreover, one can see both these bands at the absorption spectrum of Cr doped ZnWO_4_ crystal (Figure 2 in Ref. [22]). In contrast, at the spectrum of ZnWO_4_ crystal without the specially introduced chromium (another line in the same graph), the longer-wavelength band is completely absent, although the band near 510 nm has an intensity comparable with that of the Cr-doped crystal.

The absorption bands near 460 and 510 nm overlap with the above-mentioned ZnWO_4_ luminescence band [22,24] and can also overlap with the luminescence bands of some rare-earths dopants. Therefore, the ZnWO_4_ coloration can worsen the scintillation and lasing properties of the crystal [25]. The particular extent of this worsening is evaluated in [10,29]. It is noted [10] that ZnWO_4_ crystal grown from ZnO and WO_3_ chemicals containing 0.15 ppm of Fe essentially reduces scintillation light yield as compared to crystal grown from the initial chemicals containing 0.08 ppm of Fe. Furthermore, increase of Fe content in the initial chemicals, up to 1 ppm, reduces the scintillation intensity of ZnWO_4_ crystal down to the level at which the light yield already cannot be measured (less than 1000 ph/MeV). Thus, the development of ways to eliminate this parasitic coloration in a ZnWO_4_ crystal is an urgent task.

One solution is to use specially purified chemicals [3,10,30], or the commercially available chemicals that have an extra-high purity grade [29] for crystal growth. This approach produces visually colorless ZnWO_4_ single crystals with rather small residual optical absorption in the visible spectral region. In fact, in [3], the use of specially purified WO_3_ with residual content of transition metals “not more than 1 ppm” and commercially available ZnO (purity 99.995%, content of Pb ≤ 10 ppm, Cd ≤ 5 ppm and less than 1 ppm of other metal ions) made it possible to grow crystals with an absorption coefficient of 0.102 cm^−1^ at λ = 460 nm.

After oxygen annealing, this absorption coefficient reduced to 0.014 cm^−1^, although the absorption intensity at λ = 370 nm doubled. However, the purification technology for WO_3_ is not disclosed by the authors in Ref. [3].

The usage of different combinations of various commercially available chemicals with different purity qualification (extra-pure, analytically-pure, chemically pure, etc.) allowed growing visually colorless ZnWO_4_ crystals by Czochralski [29]. Unfortunately, the detailed information about impurity compositions of the initial chemicals and the grown crystals were not provided in [29]. The optical absorption spectra was presented at the scale of optical densities, making it difficult to compare the residual absorption values achieved in the crystals with those from other authors or the results presented below.

We only found one study [30] that described the technology for deep purification of ZnO and WO_3_, which enabled the growth of visually colorless ZnWO_4_ crystals. The purification of ZnO was based on its dissolution in HCl, followed by the extraction of the formed Fe^3+^ and Cr^6+^ complexes by organic solvents, and removal of the residual HCl by distillation. Then, Zn(OH)_2_ was precipitated from the purified ZnCl_2_ solution by ammonia. The precipitate was dissolved in HNO_3_ to remove traces of the residual chloride ions. Finally, ZnO was obtained from Zn(NO_3_)_2_ by drying and annealing at 1000 °C for 2 h.

The purification of WO_3_ was based on slow precipitation of H_2_WO_4_ from Na_2_WO_4_ aqueous solution by H_2_O_2_ in the presence of HNO_3_ at heating, followed by quick filtering and washing of the precipitate with a HNO_3_—NH_4_NO_3_ mixture. The resulting precipitate was dissolved in an ammonia solution and heated to crystallize ammonium paratungstate, which was then transformed into lemon-yellow colored WO_3_ by annealing at 750 °C for 2 h. Thus, a complex, multi-step purification technology for both chemicals was used.

The ZnWO_4_ crystal that was grown from the purified ZnO and WO_3_ chemicals exhibited a coefficient of parasitic optical absorption of ~0.24 cm^−1^ at λ = 460 nm [30].

Another approach is the use of special co-dopands (Nb^5+^, Ce^4+^, Ag^+^ [10] and Ta^5+^, Sb^5+^ [22]) to completely remove crystal coloration (absorption coefficient at λ = 460 nm is practically equal to zero [30]). The above co-dopants promote complete iron oxidation to its trivalent state. The absorption band of the Fe^3+^ ion is believed to lie almost completely in UV spectral range and, hence, it does not influence the crystal coloration. However, these special co-dopants can influence some other important properties of ZnWO_4_, including behavior of the main dopants in the crystal. Therefore, these co-dopants should be used with caution. In addition, the co-doped crystals have lower scintillation light yield [10] and resistance to radiation damage [20] compared to colorless undoped ZnWO_4_ crystals grown from specially purified chemicals.

In the present research, we present development of new ZnO and WO_3_ deep purification techniques (simpler than those disclosed in [30]) on ZnWO_4_ crystal growth through the use of the purified chemicals (hereafter, “pure” crystal) and on the measurement of the actual accidental impurities compositions, both of the initial chemicals and of the grown crystal. Additionally, we compare the spectroscopic properties of our crystals to those measured for a similar ZnWO_4_ crystal, grown at the same conditions, but using commercially available 5N ZnO and WO_3_ initial chemicals (hereafter, “crystal-etalon”).

## 2. Materials and Methods

### 2.1. Impurity Determination by ICP-MS

The initial WO_3_ powder (REACHEM JSC, Moscow, Russia), with a chemical purity of 99.97 wt%, was put into a polypropylene reactor. Then, an ammonia aqueous solution (10%) with a chemical purity of 99.98 wt% was added to the powder. The mixture was heated to 75 °C with continuous stirring for 2 h to dissolve the WO_3_. During this process, a concentrated ammonia solution was slowly added into the reactor by a peristaltic pump in order to provide the necessary NH_3_ concentration to allow the reaction of ammonium paratungstate formation to proceed. This stepwise addition of ammonia allowed keeping for optimal concentration in the reactor without essential evaporation losses.

The obtained solution was then filtered through a polyestersulphonic membrane (0.1 μm pores size), with help of peristaltic pumping. The filtered solution was slowly evaporated to 60% of its initial volume in the reactor. The ammonium paratungstate crystals were precipitated and grew during the evaporation. The obtained crystals were then separated from the solution by a nutch filtration procedure. The crystals were washed by deionized water and dried under vacuum. The deionized water used here and in all below described processes was produced by an Aqua-MAX-Ultra 370 Series setup (Young Lin Instruments Co., Ltd., Seoul, Republic of Korea). The obtained water had a purity of 99.999999 wt% (based on 68 elements) and had an electrical resistance of 18.2 MΩ × cm.

The dried ammonium paratungstate crystals were then loaded into a fuzed silica glass container for thermal decomposition. The container was put into the reactor, which, in turn, was put into a resistive furnace, where thermal decomposition was carried out according to the following step-scheme: heating to 200 °C at a rate of 200 °C/h; heating to 350 °C at 30 °C/h with 1 h exposure at 350 °C; heating to 500 °C at 100 °C/h; heating to 700 °C at 400 °C/h with 1.5 h exposure at 700 °C; and finally, spontaneous cooling to room temperature by switching off the furnace power supply. The reactor was purged by filtered air throughout the entire thermal treatment. After the cooling, we extracted the purified tungsten oxide (VI) powder in a glove box.

Impurities concentrations in both the obtained WO_3_ powder and the commercially available 5N WO_3_ chemical were analyzed using inductively coupled plasma mass spectrometry (ICP-MS). The samples for the analysis were transferred from the solid phase into the liquid form by dissolution in an ammonia aqueous solution (99.999 wt%). The obtained solutions were placed into polypropylene tubes (PP) and diluted by deionized water for further analysis. Analytical measurements were performed on a NexION 300D inductively coupled plasma mass spectrometer (PerkinElmer Inc., Waltham, MA, USA) using the kinetic energy discrimination (KED) mode. The TotalQuant method [31] was used for determination of 65 chemical elements concentrations. The standard solutions (PerkinElmer Inc.) were used for calibration. 

ZnWO_4_ phase purity was approved by XRD analysis (Figure S1). The pieces of ZnWO_4_ crystals, ground into the powders in an agate mortar, were dissolved in 10 mL of hydrofluoric acid (99.99999 wt%) purified by a BSB-929-IR surface distillation system, (BERGHOFF GmbH&Co., Wenden, Germany) using microwave digestion at polytetrafluoroethylene autoclaves (DAP-100, PTFE, BERGHOFF GmbH&Co., Wenden, Germany) at 200 °C for 180 min. The microwave treatment was carried out using a SPEEDWAVE unit (BERGHOFF GmbH&Co., Wenden, Germany). Then, the resulting solution was transferred to a PP test tube and diluted with water. The solution was then analyzed using inductively coupled plasma mass-spectrometry (ICP-MS). The remaining steps of ICP-MS analysis of ZnWO_4_ crystals after the dissolution were the same as those used for WO_3_ and ZnO analysis.

**Figure 1:**
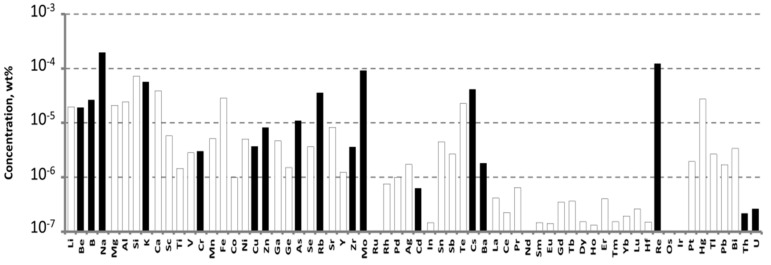
Impurities concentrations determined by ICP-MS in the purified WO_3_. Hereafter, the empty bars indicate the limits of determination (LD) of ICP-MS analysis.

The impurity composition of the purified WO_3_ in accordance with ICP-MS analysis is presented in Figure 1.

The purified WO_3_ had impurities concentrations that were mostly below the limits of determination (LDs) for these elements. The main accidental impurities in the purified WO_3_ were molybdenum, rhenium, and alkali ions. Their concentrations approached—or even exceeded—10^−4^ wt% (1 ppmw). Cr and Fe are considered as the main contributors of ZnWO_4_ crystal coloration: the Cr concentration was 2.9 × 10^−6^ wt% (0.029 ppm), and Fe concentration was below the LD of 3 × 10^−5^ wt% (0.3 ppm) (for details, see Table S1).

**Figure 2:**
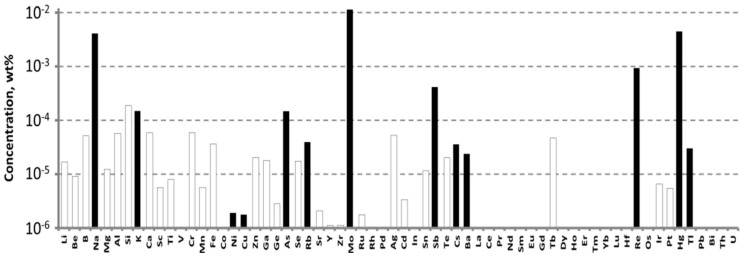
The impurity composition of the WO_3_ 5N from “LANHIT, Ltd.” measured by ICP-MS.

To grow a crystal-etalon, we utilized the commercially available WO_3_ chemical from “LANHIT, Ltd.” (Moscow, Russia), for which the declared purity was 5N. The impurity composition of this chemical, measured by ICP-MS, is presented in Figure 2.

The main impurities that were revealed here were, again, molybdenum, rhenium, alkali ions and arsenic: their concentrations were much higher in this chemical, reaching values of 10^−2^ wt% (100 ppm). The concentrations of Cr and Fe ions were below the LDs: 0.8 ppm for chromium and 0.6 ppm for iron. 

The metallic Zn (99.99 wt% LANHIT Ltd., Moscow, Russia) used for the purification was preliminary etched for 3 min in extra-pure HNO_3_ (Komponent-Reactive Ltd., Moscow, Russia) and diluted to a concentration of 15%. Then, Zn was additionally purified by vacuum distillation.

The purified metallic Zn was loaded into a Teflon container, where it was completely dissolved in concentrated extra-pure HNO_3_. The obtained solution was diluted by deionized water. Then, the Teflon container, containing the solution, was heated to 75 °C. An aqueous ammonia solution (15%) was added with continuous stirring by a polypropylene drip funnel until pH = 5. The pH was monitored with an OHAUS Starter 300 pH-meter equipped with a ST320 pH-electrode (OHAUS Europe GmbH, Nänikon, Switzerland). Aging of the formed precipitate was conducted for 2 h. The obtained suspension was filtered through a glass-fiber filter with a pore size of 1 μm. Then, a diluted, aqueous ammonia solution was added to the filtrate until a pH value of 7.5 was reached. The obtained solution was aged for 24 h with continuous stirring at 75 °C. The precipitate was filtered thoroughly and washed with deionized water. This procedure was repeated five times. Finally, the sediment was dried at 80 °C for 24 h.

The dried precipitate was loaded into a fused silica glass crucible for thermal decomposition. The crucible was put into the reactor, which, in turn, was loaded into a furnace, where annealing was performed at 600 °C in a pure oxygen atmosphere (P_O_2__ = 180 Torr) for 4 h. 

**Figure 3:**
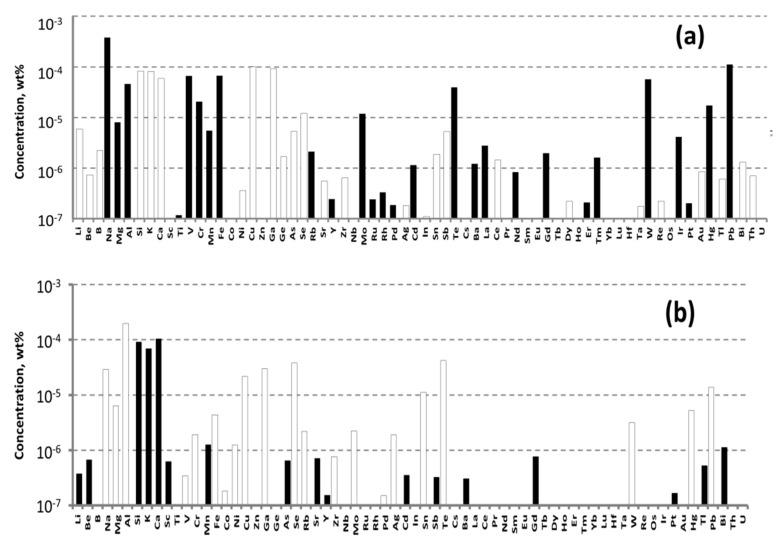
Impurities concentrations determined in the commercial (**a**) and purified (**b**) ZnO by ICP-MS analysis.

The concentrations of impurities in the purified ZnO were measured by ICP-MS analysis. The powder was transformed into a solution for the analysis by dissolving it in high purity nitric acid (99.999995 wt%), which was obtained in a BSB-929-IR surface distillation unit (BERGHOFF GmbH&Co., Wenden, Germany). The analysis was carried out in the same method as sated for WO_3_ (see above). The results of the analysis are shown in Figure 3.

The main accidental impurities in purified ZnO were aluminum, barium, sodium, and boron. According to the available literature data, these materials do not substantially influence ZnWO_4_ crystal coloration. Their concentrations approached, or even exceeded, 10^−4^ wt% (1 ppmw). Cr concentration was 4.45 × 10^−6^ wt% (twice as larger than that in the purified WO_3_), whereas Fe concentration was 2.9 × 10^−5^ wt% (0.29 ppm) (for details see Table S2).

To grow a crystal-etalon, we utilized the commercially available ZnO chemical COA-ZnO-5N from Anhui Toplus Impex Co., Ltd., (Shushan District, Hefei, China). According to the ICP-MS measurements (Figure 3a, Table S4), the Fe concentration was 6.69 × 10^−5^ wt% and Cr concentration was 2.07 × 10^−5^ wt%; these values were nearly an order higher than the values for the purified ZnO.

### 2.2. Crystal Growth

The ZnWO_4_ single crystals were grown from the two above-described sets of ZnO and WO_3_ chemicals with the Czochralski technique and a “Kristall-2” growth setup. To prepare the charges for the crystal growth, the initial chemicals were dried at 700 °C before being weighed. The required amounts of the chemicals were weighted with an Adventurer AX523 electronic analytical balance (OHAUS Corp., Parsippany, NJ, USA), which has an accuracy of 0.001 g. They were then put into new polypropylene containers and thoroughly mixed using a Multi RS-60 mixer-rotator (BioSan, Riga, Latvia). The obtained mixtures were put into new corundum crucibles with covers and calcined at 700 °C for 5 h in an EKPS-10/1250 SPU 4107 muffle furnace (Allkhim Ltd., Belgorod, Russia) to complete a solid phase synthesis of the compound.

**Figure 4:**
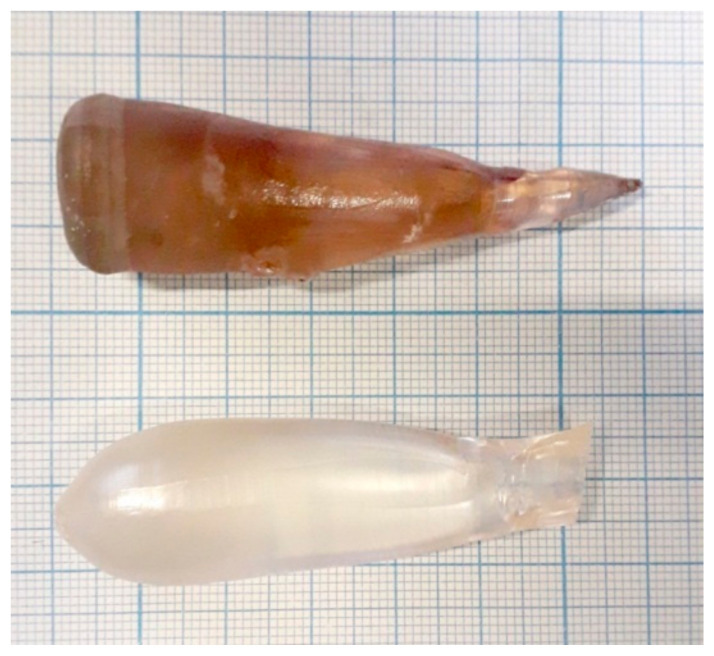
Photos of the grown crystals: the crystal-etalon (**top**) and the “pure” crystal grown from the purified ZnO and WO_3_ chemicals (**bottom**).

The growth of both crystals was performed in air from the same thoroughly pre-washed Pt crucible, which has a diameter of 40 mm diameter and a height of 35 mm. New, preliminary annealed ZrO_2_ and Al_2_O_3_ ceramic thermal shields were used (the same set for both samples). A new Pt wire was used as a seed. We did not use a ZnWO_4_ single-crystalline seed in order to avoid contamination of the melts from the seed. The pulling/rotation rates were 2 mm/h and 6 rpm, respectively. After finalizing the growth process and detaching a crystal from the melt, it was slowly (8 K/h) cooled down to room temperature in order to minimize crystal cracking. The additional post-growth annealing of the grown crystals was performed in air in a muffle furnace for 15 days at 800 °C. The heating and cooling rates were maintained at 10 K/h to avoid thermal shocks. Photos of the grown crystals are presented in Figure 4.

The coloration of the grown crystals differed drastically: the crystal-etalon had a pronounced beige brown coloration typical of ZnWO_4_ single crystals grown from the usual commercially available chemicals (see, for example, the photos given in [1,2,6,10]). Meanwhile, the “pure” crystal was almost colorless.

**Figure 5:**
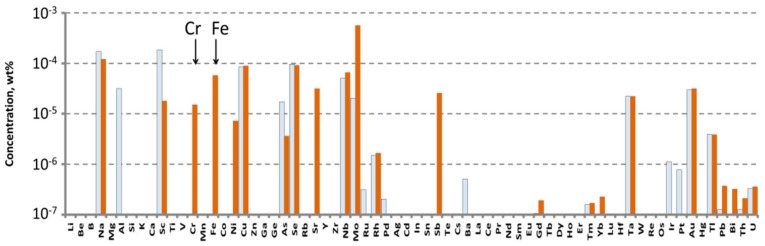
Impurities concentrations in the crystal-etalon (orange bars), and the “pure” crystal (white bars) determined by ICP-MS.

We measured the actual impurity compositions of both crystals using ICP-MS analysis (Figure 5, Table S3). 

The main accidental impurities in the crystals appeared to be

- Sodium: the revealed concentrations were in the range of 1–2 ppm. These values were comparable in both crystals and close to the Na^+^ concentrations in the specially purified ZnO and WO_3_, although they were much lower than the Na^+^ concentration in WO_3_ from «LANHIT Ltd».
- Scandium: the revealed concentration of Sc in the “pure” crystal is 2 ppm. This value is one order of magnitude higher than in the crystal-etalon. Note that this element was not found in the initial chemicals.
- Selenium, Niobium, Tantalum: both samples contained comparable concentrations of these elements: almost 1 ppm, 0.5–0.6 ppm, and ~0.2 ppm for Se, Nb and Ta, respectively. Again, none of these elements were found anywhere in the initial chemicals.
- Molybdenum: the concentrations of this impurity are 0.2 ppm in the “pure” crystal and almost 6 ppm in the crystal-etalon. These concentrations were a factor of 5–15 less than Mo concentrations in the corresponding WO_3_ initial chemicals.

In the crystal-elation, the concentrations of the most dangerous pollutants, Fe and Cr, were

- Chromium: 0.15 ppm (two times more than the measured value in WO_3_ from «LANHIT Ltd»);
- Iron: 0.6 ppm (comparable with the concentrations of this impurity in commercial ZnO).

The concentrations of Fe and Cr in the “pure” sample are below the LD of ICP-MS for these elements (0.017 and 0.4 ppm, respectively).

### 2.3. Spectroscopic Measurements

Plates with thicknesses of 1.5 mm and polished facets were made from the grown boules. The facets were parallel to the (010) crystallographic plane, which is a well-expressed cleavage plane for the ZnWO_4_ crystal. This plane is perpendicular to the N_p_ principal axis of the optical indicatrix of this crystal. Therefore, the optical absorption spectra measured through these plates (perpendicular to the polished facets) without any polarizing elements give the partially polarized (E‖N_m_ + E‖N_g_) spectra. Such spectra were measured with the Cary 5000 spectrophotometer (Agilent Technologies Inc., Santa Clara, CA, USA) in the spectral range of 300–1000 nm with a step of 1 nm.

Stationary photoluminescence spectra of the crystals were measured with three types of equipment at different UV-excitation wavelengths.

1. The measurement was performed using a Fluorolog FL3-22_1447C-2911-FL spectrofluorimeter (Horiba Jobin Yvon, Portland, OR, USA) in the 370–750 nm spectral range with a scanning step of 1 nm. The excitation wavelength was 312 nm. The same device was also used to measure fluorescence excitation spectra (265–450 nm excitation wavelengths with 1 nm step; the monitoring wavelength was 486 nm), as well as for the luminescence decay kinetics measurements (the excitation wavelength was 312 nm, the monitoring wavelength was 481 nm).

2. The next measurements were done with an experimental stand consisting of the excitation source (four UVTOP^®^ LEDs (Sensor Electronic Technology Inc., 110 Atlas Court, Columbia, SC, USA) with peak wavelengths of 260 nm, 285 nm, 315 nm, and 355 nm), MDR-2 monochromator (LOMO, former USSR), FEU-83 photomultiplier tube (former USSR), and the analog-to-digital converter, coupled with a PC.

3. FSD-9 mini-spectrometer (LLC Optofiber, Moscow, Russia). A LED with a peak wavelength of 287 nm was used as the excitation source. The same mini-spectrometer was also used to measure the X-ray excited luminescence spectra. An X-Ray tube with a chromium anode was used. The accelerating voltage was 30 kV; and the current was 30 μA.

Pulsed cathodoluminescent measurements were carried out using a GIN-400 electron accelerator as an excitation source. The electron pulse duration at half-width was 12 ns. The average energy of accelerated electrons was approximately 250 keV. The luminescence decay kinetics were recorded on a FEU-84-6 photomultiplier tube using an MDR-3 monochromator (spectral range 200–2000 nm, linear dispersion 2.4 nm/mm) and a digital oscilloscope, LeCroy 6030 (350 MHz) (LeCroy Corporation, New York, NY, USA).

Integral cathodoluminescence spectra were recorded with an AvaSpec-2048 fiber-optic spectrometer (340–1100 nm) (LOKAMED Ltd., St. Petersburg, Russia).

All the spectroscopic studies were carried out at T = 300 K.

## 3. Results and Discussion

### 3.1. Optical Absorption

**Figure 6:**
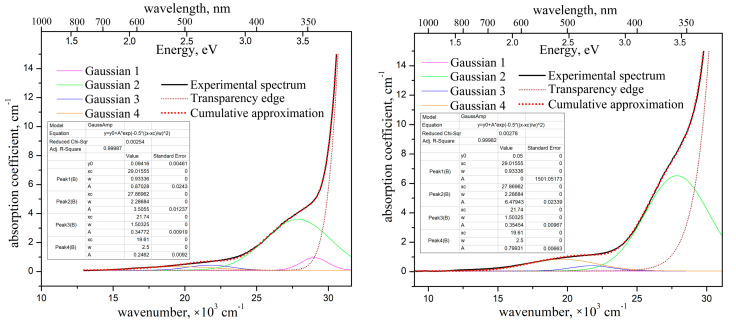
Optical absorption spectra of the “pure” ZnWO_4_ crystal (**left**) and of the crystal-etalon (**right**), and their decomposition onto the elementary Gaussians. Polarization E‖N_m_ + E‖N_g_, T = 300 K.

The optical absorption spectra of both studied ZnWO_4_ crystals are shown in Figure 6. Their decomposition into elementary Gaussians are also shown.

The absorption spectra of the ZnWO_4_ crystals are quite typical, similar to those published in [22,25]. The absorption coefficient of the “pure” crystal is ~0.65 cm^−1^ near 450 nm. This value is lower than the one reported in [22] (0.84 cm^−1^), but still higher than those published in [30] (~0.24 cm^−1^) and in [3] (0.014 cm^−1^). The absorption coefficient of the crystal-etalon at the same wavelength is about 1 cm^−1^.

We decomposed the absorption spectra into elementary Gaussians using the Microcal^®^Origin^®^ 8.0 software. We applied the same set of the Gaussians (the same combinations of peak positions $x_{i}^{c}$ and FWHMs $\omega_{i}^{}$) for both crystals. In order to approximate the broad weak shoulder at 16,000–24,000 cm^−1^ (450–550 nm), we used the sum of two separate Gaussians. The values of peak positions $x_{3}^{c}$ and $x_{4}^{c}$ of these two Gaussians have been fixed (without any variations) on the peak positions of Fe^2+^ and Cr^3+^ absorption bands, at wavelengths of 460 nm (21,740 cm^−1^) and 510 nm (19,610 cm^−1^), respectively, according to the literature data [10,22,24]. The peak position of the third, much stronger and slightly narrower Gaussian, $x_{2}^{c}$, was varied. The position was optimized in the frame of the approximation to be ~27,870 cm^−1^ (359 nm). This value is close to the literature data on Fe^3+^ absorption peak position [26,29]. The values of $\omega_{i}^{}$ for all Gaussians were also varied and have been optimized to the frame of the approximation (see insets in Figure 6).

It appears that the use of the above three Gaussians does not suffice to create an adequate approximation of the absorption spectrum of the “pure” crystal, in contrast to the spectrum of the crystal-etalon. Thus, we had to introduce a fourth Gaussian, for which the value of $x_{1}^{c}$ was optimized to be 29,016 cm^−1^ (345 nm). The intensity of this Gaussian appeared to be almost 1 cm^−1^. Figure 5 shows that only Sc presents in the “pure” crystal with the pronounced concentration, exceeding the one for the crystal-etalon by an order of magnitude. The band at 345 nm perhaps corresponds to a point defect in the lattice formed during the introduction of heterovalent Sc^3+^ into the crystal.

Comparing the elementary optical absorption bands of Fe^3+^ and Cr^3+^ in the studied crystals, one should note that intensities of these bands in the crystal-etalon exceed the ones in the “pure” crystal by factors of 1.9, and 3.2, respectively. Meanwhile, Fe^2+^ content appeared to be nearly the same in both crystals. It is possibly caused by some crystallochemical or thermodynamic mechanism of stabilization for Fe^2+^ concentration in ZnWO_4_ crystal. This mechanism could be related to the fact that Zn^2+^ → Fe^2+^ substitution in the lattice is isovalent, contrary to that for Zn^2+^ → Fe^3+^ and Zn^2+^ → Cr^3+^ substitution.

### 3.2. Photoluminescence

**Figure 7:**
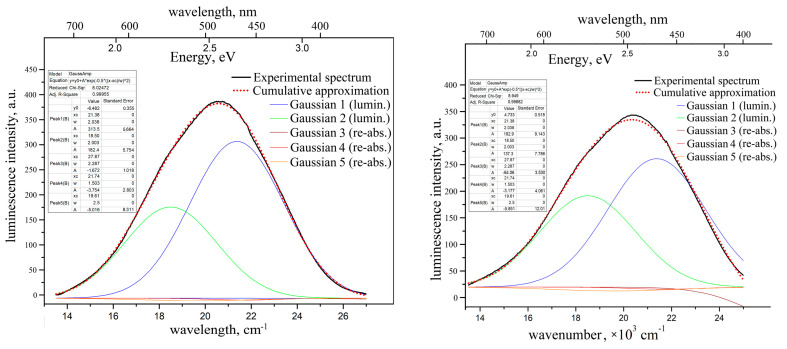
Unpolarized photoluminescence spectra of “pure” ZnWO_4_ crystal (**left**) and of the crystal-etalon (**right**), and their decomposition onto the elementary Gaussians. λ_ex_ = 312 nm, T = 300 K.

The photoluminescence spectra of the ZnWO_4_ crystals are presented in Figure 7.

The spectra obtained in our research by two other ways look very similar to those presented in Figure 7. The luminescence range and the peak positions also resemble those published earlier [10,24,25,29,32]. One can see the following.

1. The luminescence intensity of the “pure” crystal is almost 1.5 times larger than that of the crystal-etalon.

2. The luminescence range and peak position for “pure” crystal are slightly blue-shifted as compared to the ones for the crystal-etalon.

3. The luminescence bands for both crystals are slightly structured with non-Gaussian shapes. From most of the published ZnWO_4_ photoluminescence spectra [10,24] it is very difficult to understand whether their shapes are close to elementary Gaussian shapes; in both papers, the spectra are built vs the wavelength (not vs the units, proportional to energy). Moreover, in [10], the spectrum is presented by a rough dashed line. In [24], the spectrum measurement step was as wide as 10 nm. In addition, we are not sure that the spectra from [10,24] are properly corrected for the spectral function of the recording devices.

The photoluminescence spectrum of ZnWO_4_ nano-powder under VUV synchrotron excitation is studied in [32] vs the quantum energies (eV). The non-Gaussian shape of the photoluminescence band presented in the paper is evident as well.

We decomposed the photoluminescence bands of both samples into the elementary Gaussians. It was found that two Gaussians are enough for adequate approximation of the “pure” crystal luminescence band: $x_{1}^{c}$ = 21,380 cm^−1^ (468 nm, 2.65 eV), $\omega_{1}$ = 2038 cm^−1^; $x_{2}^{c}$ = 18,500 cm^−1^ (540 nm, 2.3 eV), $\omega_{2}$ = 2003 cm^−1^. The intensity of the former Gaussian is almost twice as large as that of the latter one. The paper, [32], also discusses the second luminescence component peaking near 2.3 eV; however, this component is only well expressed for Ni-doped ZnWO_4_ powder in this paper.

In order to evaluate the reabsorption influence on the photoluminescence bandshape, we have also introduced the main three optical absorption Gaussians into the approximation model with “negative” intensities. The results of the approximation show that influence of the reabsorption is negligible for the “pure” crystal (see Figure 7).

The luminescence band for the crystal-etalon has been decomposed into the same five elementary Gaussians (two “positive” luminescence peaks and three “negative” reabsorption peaks). The intensities ratio between the first and second luminescence Gaussians is ~1.4. Both peaks are significantly smaller in the crystal-etalon than the ones in “pure” crystal. The influence of two longer-wavelength reabsorption peaks (with $x_{4}^{c}$ = 21,740 cm^−1^ and $x_{5}^{c}$ = 19,610 cm^−1^) is still insignificant. The effect of the reabsorption of the band with $x_{3}^{c}$ = 27,870 cm^−1^ is much more pronounced than for the “pure” sample (see Figure 7). This fact explains the slight red shift of the luminescence band of the crystal-etalons.

We failed to find detailed studies of the effect of reabsorption on the shape and/or position of the peak of ZnWO_4_ luminescence band in the available literature. In [10], it was shown that the doping of a crystal with iron reduces the overall luminescence intensity. In addition, a table, given in [24], lists the exact positions of the luminescence peaks and half-widths for Fe:ZnWO_4_ crystals with various methods of doping with iron and/or co-doping with Li^+^. However, the article does not provide any comments/explanations on the contents of this table, nor the optical absorption spectra of crystals. Thus, from the presented data, it is very difficult to understand how these doping features affect the luminescence band or the intensities of the main spurious absorption bands.

As noted above, the reabsorption of luminescence by the optical absorption band at 27,870 cm^–1^ (359 nm) explains the red shift of the luminescence band of the crystal-etalon well. However, this does not explain the significant decrease in the intensity of its luminescence (Gaussians 1 and 2) in comparison with a “pure” crystal. This decrease can be explained, either by partial parasitic absorption of the exciting light, or by the presence of some luminescence quenching centers.

**Figure 8:**
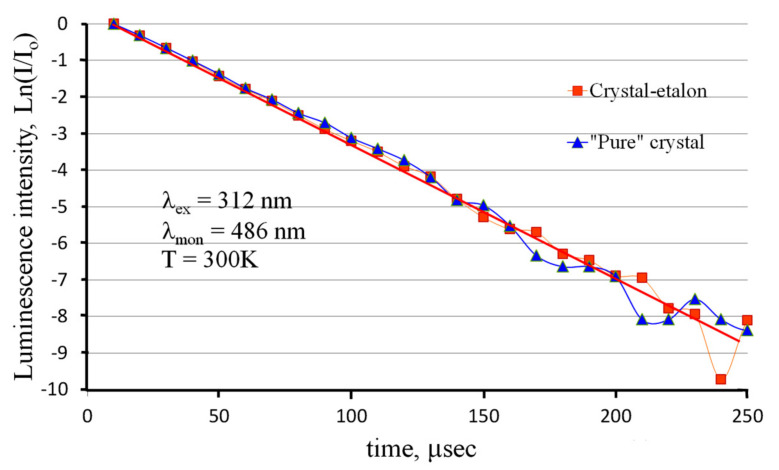
Photoluminescence decay kinetics for the studied ZnWO_4_ crystals.

In order to confirm or reject the latter version, the photoluminescence decay kinetics was measured for both studied ZnWO_4_ crystals. The results are presented in Figure 8.

One can see that the luminescence decay kinetics of both crystals can be fitted with a single-exponential function with a lifetime of 29.2 μs. This result agrees well with previously published results [12]. The decay kinetics are the same within the measurement error for both samples. This means that only one type of luminescence center exists in crystals under UV excitation. Note that luminescence monitoring was carried out at a wavelength of λ_mon_ = 486 nm (20,580 cm^−1^, 2.55 eV), where the contributions of both luminescence Gaussians are comparable (see Figure 7). If these two luminescence Gaussian components had different lifetimes, the acquired decay kinetics would be poly-exponential in these conditions of measurements. The observed single-exponential kinetics mean that both Gaussians have the same lifetime. Therefore, these Gaussians probably originate from the same type of luminescence centers. The splitting of the emission band can be explained by the splitting of either the final energy state of the luminescent transition or the initial level of the transition.

The authors of [12] considered the structure of [WO_6_] cluster to be responsible for the ZnWO_4_ luminescence. They noted that this cluster has two sets of W–O bonds with different lengths. This should result in splitting the energy of self-trapped exciton based on the [WO_6_] cluster into two separate components. Luminescent transitions from these two components of the excited energy level should lead to two pronounced luminescence bands. The two observed luminescence Gaussians possibly correspond to the transitions from these two excited energy levels.

However, some ideas make this version questionable: the energy gap ΔE between the Gaussians maxima is almost 2900 cm^−1^: this is sufficient for fast transfer of the energy of the excited state from a higher energy level to a lower one by means of nonradiative multiphonon relaxation. This also takes into account that both excited energy states are vibronic ones. However, such a value of ΔE is too large for the thermal coupling of these two energy states, which could lead to a rapid mutual exchange of energy between these two states and to equalization of the decay lifetimes between them. Thus, it is most likely that luminescence only occurs from the lowest component of the split excited state with one luminescence decay constant, while the splitting of the luminescence band is due to other reasons.

Other possible explanations of the local ZnWO_4_ luminescence band near 2.3 eV are recombination of *e–h* pairs localized at oxygen-atom-deficient tungstate ions [33,34] or distorted WO_6_ octahedra [35]. However, in this case, the luminescence component near 2.3 eV should have a decay time that is different from the one of the main luminescence peak: this is not the case (see Figure 8).

**Figure 9:**
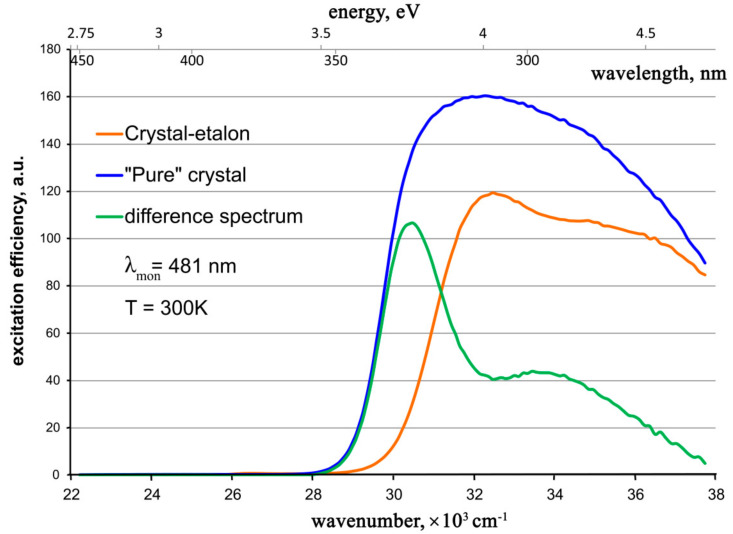
Photoluminescence excitation spectra for the studied ZnWO_4_ crystals and the difference spectrum.

The observed single-exponential luminescence decay kinetics also indicate the absence of any mechanisms of nonradiative interaction between luminescence centers and random impurity ions in crystals. Consequently, a certain decrease in the luminescence intensity of the ZnWO_4_ standard crystal is probably due to some mechanisms of a decrease in the excitation efficiency in this crystal compared to a “pure” crystal. To clarify the situation, the photoluminescence excitation spectra of the studied ZnWO_4_ crystals were measured (Figure 9).

It is seen that

1. The excitation spectra for both crystals are broad and non-elementary bands. Our attempts to adequately approximate the excitation band of the “pure” crystal, even by three Gaussians, failed. Apparently, at least four Gaussians are necessary for such an approximation. Therefore, the excitation mechanism of the [WO_6_]-cluster responsible for the photoluminescence of the ZnWO_4_ crystal [11,12] is rather complicated. It includes several possible electron transitions that perhaps occur in several optical centers.

2. None of the absorption bands in the visible or near UV spectral regions assigned to Cr^3+^, Fe^2+^, or Fe^3+^ [2,22,26,29] take part in the luminescence excitation.

3. The longer wavelength shoulder of the luminescence excitation spectrum for the “pure” crystal coincides well with the line of the “transparency edge” of the optical absorption spectrum, determined within the approximation of this spectrum (Figure 6). It is indicated with a thin brown dotted line. Thus, this “transparency edge” line is probably the longer wavelength shoulder of the charge-transfer absorption band of the [WO_6_] cluster.

4. In the difference between the excitation spectra for the “pure” crystal and the crystal-etyalon, one can clearly see the superposition of two bands close to the Gaussian shape: a stronger and narrower band with a maximum of ~30,600 cm^–1^ (327 nm, 3.79 eV), as well as a weaker and broader band with a maximum at ~33,600 cm^−1^ (298 nm, 4.16 eV). Two assumptions are possible: (1) These bands correspond to additional excitation centers present in the “pure” crystal and absent or greatly reduced in the crystal-etalon. After absorbing the photons of the exciting light, these centers transfer the energy of their excited state to the [WO_6_] clusters; (2) These bands belong to parasitic absorption centers in the crystal-etalon. They are probably associated with accidental impurities in the crystal.

The obtained luminescence excitation spectrum for a “pure” crystal resembles the low-energy part of the excitation spectrum measured earlier for the ZnWO_4_ nano-powder [32], while the excitation spectrum for the crystal-etalon closely resembles that measured earlier for the Fe,Li:ZnWO_4_ crystal at 77 K, in which iron was introduced in a metallic form [24].

### 3.3. X-ray Luminescence

**Figure 10:**
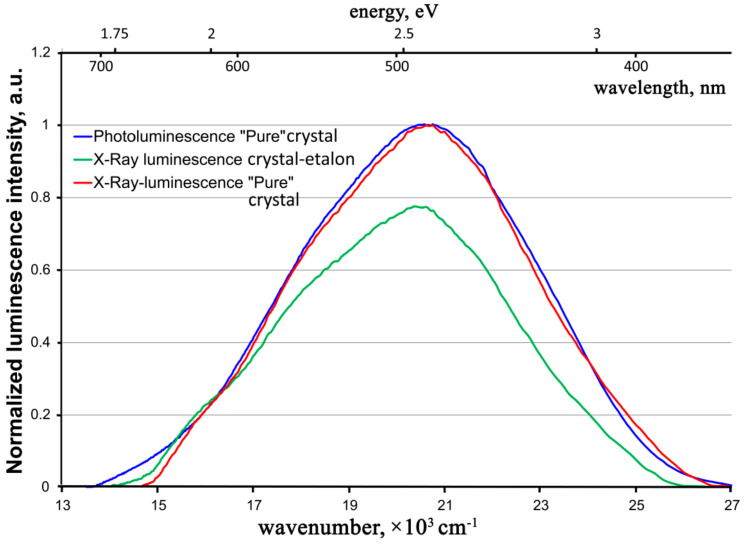
X-Ray luminescence spectra of the “pure” ZnWO_4_ crystals in comparison with its photoluminescence spectrum.

The X-ray luminescence spectra of the crystals resemble photoluminescence ones (Figure 10). 

In fact, the contour of the X-ray luminescence band of a “pure” ZnWO_4_ crystal practically coincides with the contour of the photoluminescence band of the same crystal. The situation is the same with the literature data on photo- and X-ray luminescence of previously studied ZnWO_4_ crystals (compare Figure 3 in Ref. [24] and Figure 4 in Ref. [10] with Figure 2 in Ref. [12] and Figure 2 in Ref. [2]). The same optical center participates in the luminescence process for both types of excitations in ZnWO_4_ crystals.

In this case, the intensity of X-ray luminescence of the “pure” crystal essentially exceeds the one for the crystal-etalon. The intensities ratio is similar to that of photoluminescence spectra (see above). It is possible that, at a certain stage, the excited state energy transformation after X-ray irradiation proceeds through the same steps as in the case of UV excitation. Consequently, at this stage in the crystal-etalon, the system loses the same part of the energy on uncontrolled impurities, both after UV and after X-ray excitation. 

### 3.4. Cathodoluminescence

**Figure 11:**
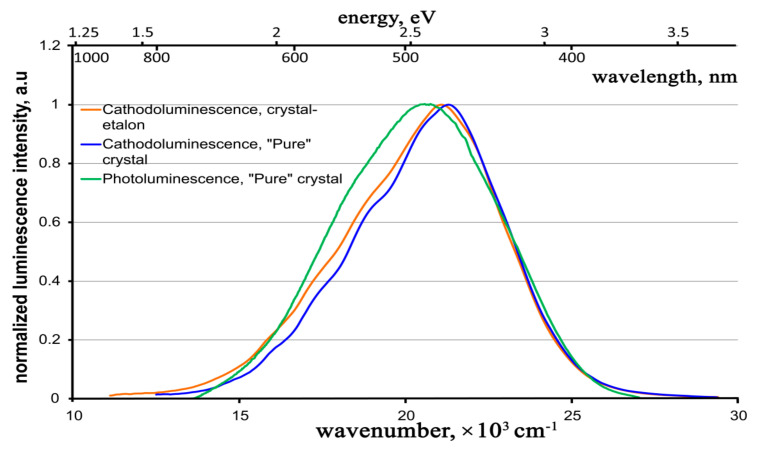
Normalized cathodoluminescence spectra for both the crystals under study and photoluminescence spectrum for the “pure” crystal.

The normalized cathodoluminescence spectra for both the crystals under study are given in Figure 11, along with the photoluminescence spectrum for the “pure” crystal.

One can see that, in contrast to the case of photoluminescence, the cathodoluminescence spectra for both studied samples have approximately the same shape, spectral ranges, and peak positions (within the measurement error). In addition, these spectra show slight differences from the photoluminescence spectra, especially in the longer wavelength region; they have a non-Gaussian shape. It is likely that the longer wavelength Gaussian, which manifests itself in the photoluminescence spectra (Figure 7), is reduced in the cathodoluminescence spectra. However, as far as we can judge from the luminescence spectra, the luminescence center is generally the same in the ZnWO_4_ crystal, regardless of the excitation method (UV, X-ray rays, electron beam).

**Figure 12:**
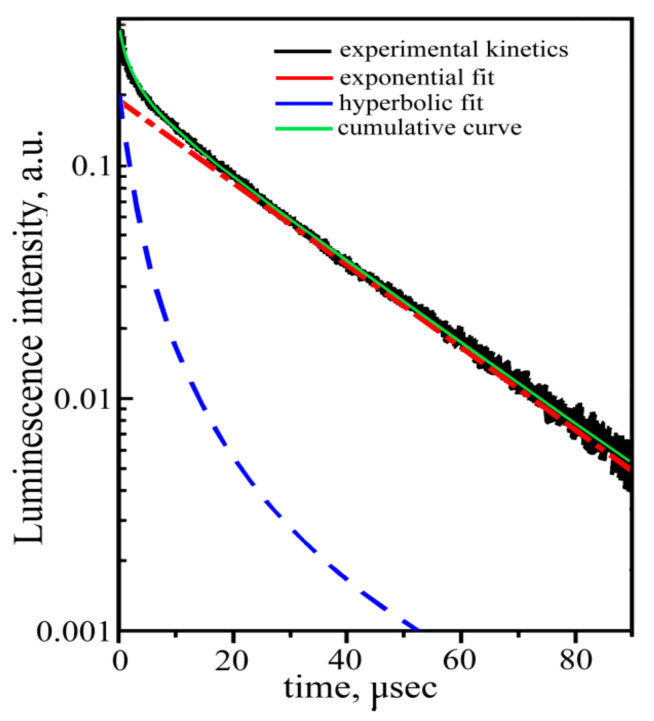
Cathodoluminescence decay kinetics of the “pure” crystal.

In this case, the cathodoluminescence intensity of the crystal-etalon is ~1.5 times higher than that of the “pure” crystal, in contrast to the ratios of the photo- and X-ray luminescence intensities. To reveal the mechanisms of cathodoluminescence, we measured the decay kinetics for both crystals under study. The revealed kinetics are practically the same for both crystals, just as in the case of the photoluminescence decay kinetics. The kinetics for a “pure” crystal are shown in Figure 12.

In contrast to the photoluminescence decay kinetics, both measured cathodoluminescence decay kinetics demonstrate the non-exponential nature of the recombination process (Figure 12). The same result was obtained earlier, in [12] for, the luminescence decay kinetics of ZnWO_4_ upon excitation by an electron pulsed beam (E ~270 KeV, *τ* = 10 ns). The measured kinetics can be fitted with the sum of single exponential and hyperbolical components: the single exponential component is responsible for the intra-center luminescence of the [WO_6_] cluster; and the hyperbolical components reflect luminescence due to electron-hole recombination:

I=I1·exp−tτ+I0(1+αt)−β

where *I* is the amplitude; and *τ* and *α* are time constants of the exponential and of the hyperbolic decay components, respectively, *β* is a hyperbola index. The approximation gives the following parameters: *τ* = 20 μs, *α* = 0.2 μs^−1^, and *β* = 2. Note that the lifetime of the exponential component of the cathodoluminescence decay kinetics substantially differ from the one for the photoluminescence decay (see above). It means that, despite the similarity of photo- and cathodoluminescence spectra, the mechanisms of these types of ZnWO_4_ luminescence are different. These mechanisms require further studies.

In [12], the cathodoluminescence decay kinetics of ZnWO_4_ were described by the sum of two exponents with the decay times. None of the exponents coincided with the lifetime of the photoluminescence decay kinetics. The authors explained that these two exponents were two types of self-trapped excitons in the [WO_6_] cluster, originating from two sets of W–O interatomic distances in this cluster. We consider the explanation given in [12] to be unsuccessful and self-contradictory.

## 4. Conclusions

In the frame of this work, new, rather simple technologies for the deep purification of ZnO and WO_3_ powders have been developed. The purified powders were used as initial chemicals for Czochralski growth of a ZnWO_4_ single crystal, which has a number of potential applications. The grown crystal has a lower concentration of accidental impurities than the ZnWO_4_ crystal-etalon grown in the same conditions, but with use of initial chemicals, 5N ZnO and WO_3_, that are commercially available. The “pure” crystal is almost colorless. In contrast to crystal-etalon, it has a considerably lower intensity of parasitic optical absorption bands in the visible and in the nearest UV spectral regions. As a result, it demonstrates stronger visible luminescence of [WO_6_]-clusters under UV- and X-ray excitations.

The results of the performed spectroscopic studies induced a number of new questions, e.g., what is the nature of the absorption band, located near 345 nm, revealed in the “pure” crystal, and why this band is absent in more dirty (in general) crystal-etalon? What is the nature of the parasitic absorption band peaking near 510 nm (the version of Cr^3+^ accidental impurity looks very doubtful)? Why does the luminescence band consist of two elementary Gaussians, but with the same decay kinetics? Why does the shape of the luminescence band under the electron beam excitation essentially differ from those under UV- and X-ray excitations? Is the luminescent center in the crystal the same for different kinds of excitations? If “yes”, why are the luminescence decay kinetics considerably (and reproducibly) different for UV- and electron beam excitations? If “no”, what is the real nature of the luminescent centers for each of these excitations, and why are the wavelength ranges and peak positions for the luminescence bands for these excitations, in principle, rather close (although, not completely identical)? Finally, what is the particular mechanism of the luminescence intensity reduction in the presence of the pronounced amounts of accidental impurities in the crystal? In contrast, why do the same accidental impurities in the crystal enhance the luminescence intensity in the case of the electron beam pulsed excitation? Answering all these questions requires additional investigations that will be performed by our group in the future.

## Data Availability

Not applicable.

## Supplementary Materials

The following supporting information can be downloaded at: https://www.mdpi.com/article/10.3390/ma16072611/s1, Table S1. Concentration of impurities in purified WO3 determined by ICP-MS analysis; Table S2. Concentration of impurities in purified ZnO determined by ICP-MS analysis; Table S3. Concentration of impurities in ZnWO_4_ crystals determined by ICP-MS analysis; Table S4. Concentration of impurities in COA-ZnO-5N chemical from “Anhui Toplus Impex Co., Ltd.”, Hefei, China, determined by ICP-MS analysis; Figure S1. XRD pattern of powdered ZnWO_4_ crystal in comparison with reference data.
