# Supplementary material for: Influence of Accidental Impurities on the Spectroscopic and Luminescent Properties of ZnWO4 Crystal
Source: Materials (Basel). 2023 Mar 25;16(7):2611. doi: 10.3390/ma16072611 (PMC10096284; doi:10.3390/ma16072611)

Supplementary

Influence of accidental impurities on the spectroscopic and luminescent properties of $ZnWO_4$ crystal

Kirill A.Subbotin, Anatolii I. Titov, Victoria A. Solomatina, Andrew V. Khomiakov, Ekaterina R. Pakina, Viktor Yu. Yakovlev, Damir T. Valiev, Marina P. Zykova, Kristina V. Kuleshova, Yana S. Didenko, Denis A. Lis, Mikhail B. Grishechkin, Sergei Kh. Batygov, Sergei V. Kuznetsov, Igor Ch. Avetissov

Table S1. Concentration of impurities in purified WO_3 determined by ICP-MS analysis.

Element		Wt%	Element		Wt%
Li	<	2.0E-05	Sn	<	4.5E-06
Be		1.9E-05	Sb	<	2.7E-06
B		2.6E-05	Te	<	2.3E-05
Na		2.0E-04	Cs		4.1E-05
Mg	<	2.1E-05	Ba		1.8E-06
Al	<	2.4E-05	La	<	4.2E-07
Si	<	7.3E-05	Ce	<	2.2E-07
K		5.7E-05	Pr	<	6.5E-07
Ca	<	3.9E-05	Nd	<	9.6E-08
Sc	<	5.8E-06	Sm	<	1.5E-07
Ti	<	1.5E-06	Eu	<	1.4E-07
V	<	2.8E-06	Gd	<	3.5E-07
Cr		3.0E-06	Tb	<	3.7E-07
Mn	<	5.1E-06	Dy	<	1.5E-07
Fe	<	2.9E-05	Ho	<	1.3E-07
Co	<	9.9E-07	Er	<	4.0E-07
Ni	<	5.1E-06	Tm	<	1.5E-07
Cu		3.7E-06	Yb	<	2.0E-07
Zn		8.1E-06	Lu	<	2.6E-07
Ga	<	4.7E-06	Hf	<	1.5E-07
Ge	<	1.5E-06	Re		1.2E-04
As		1.1E-05	Os	<	9.7E-08
Se	<	3.6E-06	Ir	<	1.0E-07
Rb		3.6E-05	Pt	<	1.9E-06
Sr	<	8.3E-06	Hg	<	2.7E-05
Y	<	1.2E-06	Tl	<	2.7E-06
Zr		3.6E-06	Pb	<	1.7E-06
Mo		9.1E-05	Bi	<	3.4E-06
Ru	<	9.8E-08	Th	<	2.2E-07
Rh	<	7.6E-07	U		2.6E-07
Pd	<	1.0E-06			
Ag	<	1.7E-06			
Cd		6.3E-07			
In	<	1.5E-07			

Sign "<" indicates the limit of determination of the element for the certain analysis.

Table S2. Concentration of impurities in purified ZnO determined by ICP-MS analysis.

Element		Wt%	Element		Wt%
Li	<	4.30E-06	Sn		1.45E-05
Be	<	6.91E-08	Sb	<	6.69E-07
B		5.89E-05	Te	<	2.78E-06
Na		1.19E-04	Cs	<	1.31E-07
Mg	<	7.67E-06	Ba		1.29E-04
Al		1.29E-04	La	<	1.20E-07
Si	<	1.85E-05	Ce	<	6.04E-07
K	<	2.85E-05	Pr	<	5.10E-08
Ca	<	3.49E-05	Nd		7.91E-07
Sc	<	1.44E-07	Sm	<	6.91E-09
Ti		4.14E-07	Eu	<	2.07E-08
V	<	1.03E-05	Gd		2.31E-06
Cr		4.45E-06	Tb	<	5.61E-06
Mn	<	7.74E-07	Dy		3.55E-08
Fe		2.90E-06	Ho	<	1.91E-08
Co	<	7.57E-08	Er		3.01E-08
Ni		4.79E-07	Tm	<	3.21E-08
Cu	<	2.64E-06	Yb		3.52E-08
Ga		1.20E-05	Lu	<	1.52E-07
Ge	<	1.77E-05	Hf	<	6.91E-08
As	<	1.08E-05	Ta	<	2.78E-08
Se	<	7.61E-06	W		7.72E-07
Rb		1.83E-06	Re		1.27E-08
Sr		3.51E-06	Os	<	1.04E-08
Y	<	1.10E-06	Ir		2.68E-08
Zr		1.10E-06	Pt	<	1.40E-06
Nb	<	3.17E-07	Au		5.40E-08
Mo	<	7.23E-06	Hg	<	1.43E-05
Ru		3.40E-07	Tl	<	2.66E-08
Rh		3.79E-07	Pb		5.17E-06
Pd		1.20E-07	Bi		4.46E-08
Ag		3.23E-06	Th	<	3.24E-08
Cd	<	3.51E-06	U	<	3.45E-09
In	<	1.04E-08			

Sign “<” indicates the limit of determination of the element for the certain analysis.

Table S3. Concentration of impurities in $ZnWO_4$ crystals determined by ICP-MS analysis.

$ZnWO_4$ "pure" crystal						$ZnWO_4$ crystal-etalon					
Element		Wt%	Element		Wt%	Element		Wt%	Element		Wt%
Li	<	2.29E-06	Sn	<	3.66E-06	Li	<	2.37E-06	Sn	<	3.78E-06
Be	<	1.26E-05	Sb	<	8.17E-07	Be	<	1.30E-05	Sb		2.56E-05
B	<	6.32E-06	Te	<	8.38E-06	B	<	6.52E-06	Te	<	8.65E-06
Na		1.72E-04	Cs	<	4.98E-07	Na		1.21E-04	Cs	<	5.13E-07
Mg	<	5.99E-05	Ba		5.10E-07	Mg	<	6.18E-05	Ba	<	5.24E-07
Al		3.17E-05	La	<	4.53E-08	Al	<	2.23E-05	La	<	4.67E-08
Si	<	2.22E-04	Ce	<	8.51E-08	Si	<	2.29E-04	Ce	<	8.79E-08
K	<	1.13E-04	Pr	<	3.84E-08	K	<	1.16E-04	Pr	<	3.97E-08
Ca	<	2.95E-04	Nd	<	1.90E-08	Ca	<	3.04E-04	Nd	<	1.96E-08
Sc		1.85E-04	Sm	<	1.90E-08	Sc		1.79E-05	Sm	<	1.96E-08
Ti	<	2.85E-06	Eu	<	2.53E-08	Ti	<	2.95E-06	Eu	<	2.61E-08
V	<	8.04E-07	Gd		1.06E-07	V	<	8.29E-07	Gd		1.92E-07
Cr	<	1.73E-06	Tb	<	1.03E-06	Cr		1.52E-05	Tb	<	1.07E-06
Mn	<	4.88E-06	Dy	<	6.32E-09	Mn	<	5.03E-06	Dy		2.04E-08
Fe	<	4.06E-06	Ho	<	3.79E-08	Fe		5.81E-05	Ho	<	3.91E-08
Co	<	1.57E-07	Er	<	5.37E-08	Co	<	1.62E-07	Er	<	5.54E-08
Ni	<	1.17E-06	Tm		1.60E-07	Ni		7.27E-06	Tm		1.70E-07
Cu		8.53E-05	Yb		1.03E-07	Cu		8.88E-05	Yb		2.28E-07
Ga	<	1.11E-05	Lu	<	7.56E-08	Ga	<	1.14E-05	Lu	<	7.80E-08
Ge	<	1.06E-06	Hf	<	2.40E-06	Ge	<	1.09E-06	Hf	<	2.47E-06
As		1.72E-05	Ta		2.23E-05	As		3.62E-06	Ta		2.24E-05
Se		9.55E-05	Re	<	1.16E-04	Se		9.09E-05	Re	<	1.39E-04
Rb	<	1.61E-06	Os	<	4.42E-08	Rb	<	1.66E-06	Os	<	4.56E-08
Sr	<	1.03E-06	Ir		1.11E-06	Sr		3.16E-05	Ir		7.72E-08
Y	<	4.41E-07	Pt		7.74E-07	Y	<	4.55E-07	Pt	<	7.85E-07
Zr	<	8.18E-06	Au		2.97E-05	Zr	<	8.44E-06	Au		3.16E-05
Nb		5.05E-05	Tl		3.92E-06	Nb		6.61E-05	Tl		3.88E-06
Mo		2.04E-05	Pb		1.28E-07	Mo		5.68E-04	Pb		3.74E-07
Ru		3.16E-07	Bi	<	8.94E-08	Ru	<	2.81E-07	Bi		3.21E-07
Rh		1.50E-06	Th		1.28E-07	Rh		1.66E-06	Th		2.12E-07
Pd		2.04E-07	U		3.28E-07	Pd	<	1.56E-07	U		3.60E-07
Ag	<	1.94E-07				Ag	<	2.00E-07			
Cd	<	4.23E-06				Cd	<	4.37E-06			
In	<	2.53E-08				In	<	2.61E-08			

Sign "<" indicates the limit of determination of the element for the certain analysis

Table S4. Concentration of impurities in COA-ZnO-5N chemical from “Anhui Toplus Impex Co.”, Ltd., China, determined by ICP-MS analysis

Element		Wt%	Element		Wt%
Li	<	5,92E-06	Sn	<	1,87E-06
Be	<	7,35E-07	Sb	<	5,26E-06
B	<	2,24E-06	Te		3,95E-05
Na		3,80E-04	Cs		1,02E-07
Mg		7,98E-06	Ba		1,22E-06
Al		4,60E-05	La		2,77E-06
Si	<	8,21E-05	Ce	<	1,44E-06
K	<	8,16E-05	Pr	<	1,96E-08
Ca	<	5,90E-05	Nd		8,33E-07
Sc		1,64E-08	Sm		4,22E-08
Ti		1,18E-07	Eu	<	3,60E-08
V		6,64E-05	Gd		1,98E-06
Cr		2,07E-05	Tb	<	7,82E-08
Mn		5,49E-06	Dy	<	2,20E-07
Fe		6,69E-05	Ho	<	1,37E-08
Co	<	9,30E-08	Er		2,10E-07
Ni	<	3,59E-07	Tm		1,62E-06
Cu	<	9,90E-05	Yb		3,15E-08
Zn		matrix	Lu		4,60E-08
Ga	<	9,31E-05	Hf		6,82E-08
Ge	<	1,68E-06	Ta	<	1,75E-07
As	<	5,30E-06	W		5,71E-05
Se	<	1,21E-05	Re	<	2,21E-07
Rb		2,10E-06	Os	<	7,35E-09
Sr	<	5,54E-07	Ir		4,13E-06
Y		2,44E-07	Pt		2,01E-07
Zr	<	6,47E-07	Au	<	8,47E-07
Nb	<	7,38E-08	Hg		1,73E-05
Mo		1,18E-05	Tl	<	6,09E-07
Ru		2,40E-07	Pb		1,12E-04
Rh		3,32E-07	Bi	<	1,31E-06
Pd		1,85E-07	Th	<	7,02E-07
Ag	<	1,81E-07	U	<	1,16E-08
Cd		1,14E-06			
In	<	1,10E-07			

Sign “<” indicates the limit of determination of the element for the certain analysis

Figure S1. XRD pattern of powdered ZnWO4 crystal in comparison with reference data

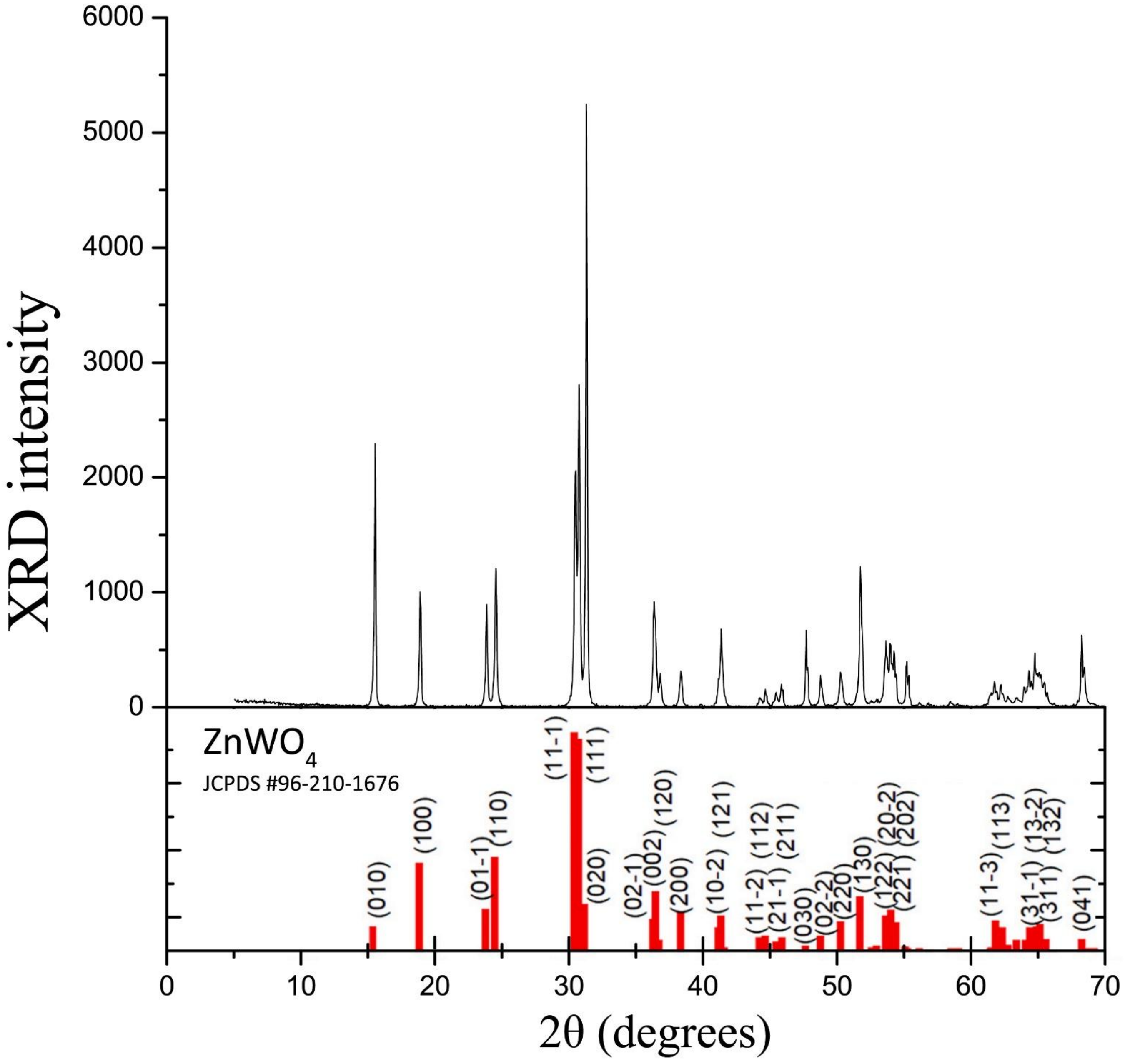

Supplement: Supplementary file 1 [file materials-16-02611-s001.zip › materials-2266399-supplementary.pdf]
